# Journal of the British Dairy Farmers’ Association for the Improvement of the Dairy Husbandry of Great Britain

**Published:** 1882-04

**Authors:** 


Journal of the British Dairy Farmers' Association for the Improvement of the Dairy Husbandry of Great Britain. Vol. I. Nos. 3 and 4. London. 8vo. pp. 142.

This journal is published by the Association for the Improvement of the

Dairy Stock and Dairy Produce of the United Kingdom. It contains several papers read at the meetings of the Association. Among the principal ones are: Shorthorns as Dairy Stock, by Finlay Dun; Abortion in Cows—an exhaustive paper on the subject, by Prof. J. Wortley Axe. In support of the theory that abortion is not contagious, he cites the following: "One of twelve cows slipped her calf about the middle of May, without any assignable cause. The fœtus and its membranes were unavoidably allowed to remain in the pasture for several hours. Three days after the event a second cow aborted; and three others followed suit, at intervals of from four days to three weeks, notwithstanding that every precaution was taken to isolate the cows as they fell, as well as in the exercise of a strict sanitary regime. From the vagina of the third case about a dram of thick, glairy discharge was taken and carefully introduced into the vagina of a cow from another stock, six months advanced in pregnancy. At the same time another portion of the same matter was inoculated into the skin of the vagina by superficial scratches. A negative result followed the experiment, and the cow completed the period of gestation, and produced a fine healthy bull calf." "From what has already been said, it will appear that the extension of abortion from one animal to another may and undoubtedly does take place, as the result of the action of the effluvium emitted from the ejected contents of the uterus on the sensorial centres; and that the prevalence of a mala [illegible]y in a herd may be explained entirely apart from the contagious influences.' Goat's Milk as Food for Infants and Invalids, by Dr. R. J. Lee, M.R.C.P.; and the Composition of Goat's Milk, by Dr. Aug. Voelcker, F.R.S. Dr. Voelcker considers goat's milk equally as nutritious as cow's milk, and givesthe following analysis of it:

| CONSTITUENTS. | PER CENT. |
|---|---|
| Water | 82.02 |
| Fat | 7.02 |
| Casein | 4.67 |
| Milk Sugar | 5.28 |
| Mineral Matter (ash) | 1.01 |
| | 100.00 |

